# gE mutations and VZV genotypes jointly predict pain relief outcomes in herpes zoster: an integrative immunologic and modeling study

**DOI:** 10.3389/fimmu.2026.1715267

**Published:** 2026-04-29

**Authors:** Ying Shi, Bin Li, Nan Li, Ai Su, Min Tang

**Affiliations:** 1 Pain Department, Shanghai Pudong New Area People’s Hospital, Shanghai, China; 2 Dermatological Department, Chongqing Three Gorges Medical College, Chongqing, China; 3 Pain Department, The First Affiliated Hospital of Chongqing Medical University, Chongqing, China; 4 College of Laboratory Medicine, Chongqing Medical University, Chongqing, China

**Keywords:** glycoprotein E, herpes zoster, immunological biomarker, pain prediction, VZV genotyping

## Abstract

**Introduction:**

Herpes zoster (HZ), caused by reactivation of varicella-zoster virus (VZV), is associated with significant pain burden and risk of postherpetic neuralgia (PHN). However, early prediction of pain outcomes remains limited due to insufficient integration of viral genotypic features and host immune responses. This study aimed to investigate the distribution of VZV genotypes, characterize glycoprotein E (gE) mutations, and evaluate their associations with immunological markers and pain outcomes in HZ patients.

**Methods:**

A total of 52 HZ outpatients from Chongqing were enrolled, and 46 samples were successfully sequenced. VZV genotyping was performed using SNP analysis of the ORF22 region, and gE gene mutations were analyzed by PCR and sequencing. Clinical data and immunological indicators, including gE antigen, gE antibody (gEAb), immunoglobulins, complement levels, and CD4+ T cells, were collected. A predictive model for pain outcomes was developed using L1-regularized regression with cross-validation.

**Results:**

Clade 2 (J-type) was the predominant genotype (76.1%), followed by Clade 3, Clade 4, and minor proportions of Clade 1 and 5. Frequent missense mutations were observed in the gE gene, particularly a T→I substitution at positions 250–291 in 67.4% of cases. The average numbers of mutations and deletions were 13.46 ± 8.07 and 5.00 ± 3.71, respectively. Immunological analysis showed detectable gE antigen and antibody levels, elevated IgG, and normal complement and CD4+ T cell levels, indicating active humoral immunity. The predictive model demonstrated good performance for identifying poor pain relief (AUC = 0.87; PR-AUC = 0.74), with gE-related variables consistently contributing to prediction.

**Discussion:**

This study demonstrates the predominance of Clade 2 VZV and identifies characteristic gE mutation patterns in HZ patients from Chongqing. The findings highlight the role of gE-related immune responses in disease progression and pain outcomes. gE may serve as a potential biomarker for clinical stratification and prediction of prolonged pain, providing insights into immunopathogenesis and personalized management of HZ.

## Graphical Abstract

**Figure:**
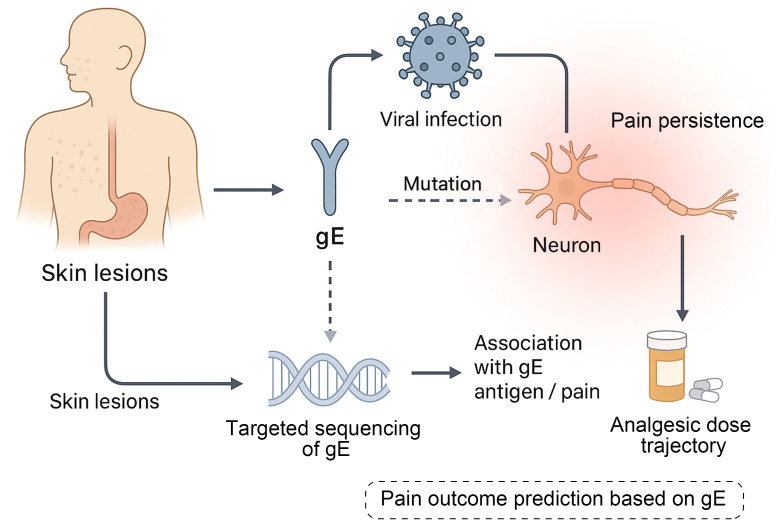
Schematic representation of the associations among VZV gE gene mutations, host immune responses, and clinical phenotypes.

## Highlights

- This study reports for the first time that Clade 2 (J-type) is the predominant VZV strain in Chongqing.
- This study finds that gE gene mutations are concentrated at positions 250-291.
- The level of gE protein is associated with lesion area and pain intensity.
- The level of gEAb shows a positive correlation with analgesic use.
- This study proposes the gE protein as a biomarker and a potential target for immune intervention.

## 1 Introduction

Varicella-zoster virus (VZV) is a highly contagious human herpesvirus. Its primary infection causes chickenpox, after which the virus remains latent in sensory ganglia. It can reactivate during periods of immune fluctuation or decline, leading to herpes zoster (HZ) (1, 2). The prevalence of latent infections in adults is extremely high, yet the biological mechanisms underlying reactivation remain inadequately understood, posing challenges to both community prevention and individual management of HZ (3). In immunocompromised individuals and some infants, chickenpox can result in severe or even fatal outcomes, indicating that the impact of VZV on susceptible hosts extends beyond dermatological manifestations (4, 5). HZ is associated with significant acute neuralgia and, in severe cases or when affecting critical areas, it can lead to risks of mortality and disability (6, 7). Postherpetic neuralgia (PHN) is one of the most debilitating complications of HZ, with an incidence rate reaching 50% to 75% among elderly patients (≥ 60 years) (8). Recent clinical observations suggest a trend towards younger onset and increased severity of PHN, further exacerbating societal and healthcare burdens (9). Current treatments focus primarily on antiviral and analgesic therapies; however, significant gaps remain in early prediction of pain outcomes and identification of high-risk patients. A fundamental issue is the insufficient understanding of the interactions between VZV latency, reactivation, neurotoxicity, and immune imbalance, which limits breakthroughs in the prevention and management of HZ and PHN.

Current evidence indicates that VZV exhibits a complex global genotype distribution pattern, characterized by ongoing gene mutations and lineage drift over time (10, 11). The geographic heterogeneity of genotypes may not only reflect the virus’s transmission routes and founder effects but also be associated with clinical manifestations, virulence profiles, and immune evasion capabilities. Glycoprotein E (gE) is one of the primary envelope glycoproteins of VZV, playing a crucial role in viral entry, budding, and cell-to-cell spread. It also serves as a key antigenic epitope for humoral immune response and vaccine protection (12, 13). Research suggests that missense mutations in the gE gene and variations in glycoprotein expression might weaken host immune recognition, thereby altering the clinical trajectory of HZ and the risk of complications (13, 14). Increased global population movement has heightened the likelihood of exogenous viral strain introduction and gene recombination events, profoundly impacting VZV’s molecular evolution and regional epidemiological patterns. In China, studies on the genotype distribution and lineage evolution of wild-type VZV across different regions remain insufficient, with systematic reports particularly lacking for Chongqing. This regional evidence gap limits understanding of clinical differences in HZ in Southwest China and the adaptability of prevention and treatment strategies, underscoring the importance of multi-regional, multi-strain research to enhance nationwide molecular epidemiological profiling.

From the perspective of host immunology, both humoral and cellular immunity play pivotal roles in controlling VZV infection and inhibiting its reactivation. Humoral immunity achieves viral neutralization through VZV-specific antibodies, although its efficacy might be influenced by antigenic drift and alterations in gE epitopes (13). The functional status of cellular immunity, particularly CD4^+^ and CD8^+^ T cells, determines the extent of suppression of latent viruses (15). Research suggests that mutations in gE may alter the recognition efficiency of humoral immunity, and different viral genotypes could affect the host’s immune threshold through structural changes, leading to variations in clinical phenotypes (13, 16). However, there is a lack of systematic evidence linking the severity of clinical pain to immunological markers; traditional indicators have limited predictive power for early pain outcomes, making it difficult to address the core question of “which patients will develop persistent pain and which will experience rapid relief.” Historically, studies on viral genetics and host immunology have remained relatively isolated disciplines, lacking integrative research that combines both with clinical phenotypes. This gap has resulted in fragmented insights into HZ mechanisms and clinical predictions. There is an urgent need to establish a comprehensive framework that spans “viral genotype—glycoprotein variation—host immune response—clinical pain phenotype” to advance from singular associative studies towards multidimensional integration and mechanistic exploration.

In this context, the present study focuses on the HZ population in the Chongqing region, aiming to systematically analyze the distribution characteristics of VZV genotypes. It seeks to uncover the mutation spectrum of the gE gene and its potential functional implications. By integrating host immunological indicators with clinical phenotypes, the study explores their roles in disease progression and pain outcomes. The specific objectives include: (1) identifying the predominant circulating strains of VZV in Chongqing and their geographical distribution; (2) systematically characterizing gE gene mutation patterns to assess potential antigenic changes and immunological associations; (3) elucidating the link between virological characteristics and pain phenotypes in conjunction with immune indicators, thereby providing insights for early risk identification. Scientifically, this research will enrich localized evidence of VZV molecular epidemiology and glycoprotein variation in China, advancing an integrated understanding of viral latency, reactivation, and pain-inducing mechanisms. Clinically, the virology-immunology-clinical phenotype framework and potential biomarkers explored in this study will offer viable pathways for early risk stratification, pain prediction, and personalized intervention for HZ, reducing the risk of complications such as PHN. Furthermore, the findings may provide regional evidence to support antigen design and immune barrier assessment for VZV vaccines, aiding in public health strategy optimization. Overall, this study holds both scientific value and clinical application potential, promising to lay a solid foundation for precise prevention and treatment systems for HZ in China.

## 2 Materials and methods

### 2.1 Study population

The study involved the selection of patients diagnosed with HZ at the outpatient department of the First Affiliated Hospital of Chongqing Medical University from November 2020 to May 2024. A total of 60 patients were initially screened, of whom 52 met the inclusion criteria and provided vesicular fluid specimens for VZV testing. Among these 52 clinical specimens, 46 were successfully sequenced and included in the final analysis. The research protocol received ethical approval from the Ethics Committee of the First Affiliated Hospital of Chongqing Medical University (Ethics Approval No: 2021-486). Before participation, all subjects were fully informed about the study and provided written informed consent.

Inclusion criteria were as follows: (1) Long-term residence in Chongqing; (2) Clinical symptoms and signs consistent with HZ diagnosis; (3) No prior antiviral treatment; (4) Voluntary participation with signed informed consent.

**Figure 1:**
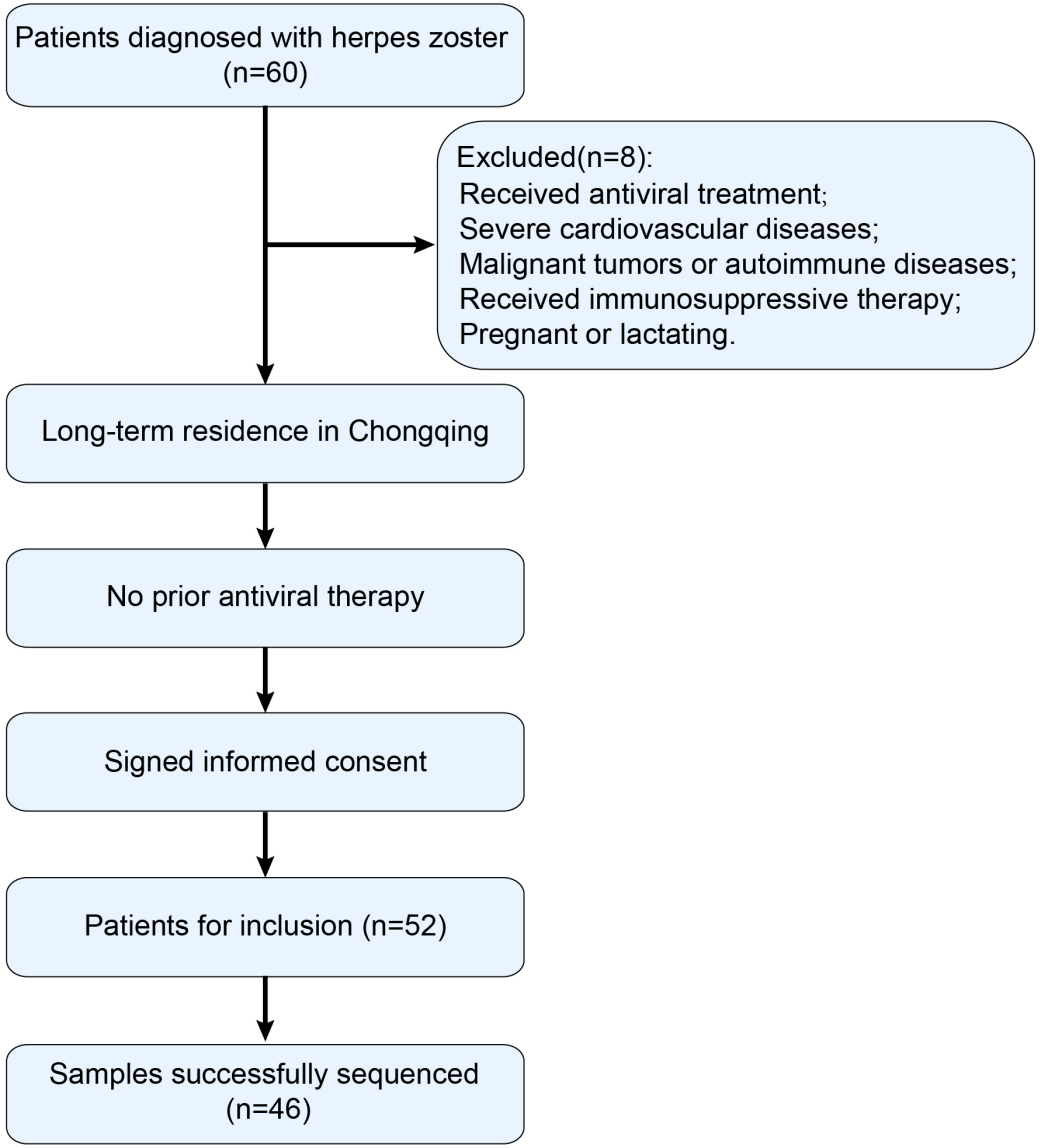
Flowchart of inclusion and exclusion criteria for patients with HZ.

Exclusion criteria included: (1) Prior use of antiviral medication; (2) Presence of severe cardiovascular diseases; (3) Diagnosis of malignant tumors or autoimmune diseases such as rheumatoid arthritis; (4) Current use of immunosuppressive agents; (5) Pregnant or lactating women (**Figure 1**).

### 2.2 Collection of clinical phenotype data

This study initially gathers fundamental epidemiological data of HZ patients, including age, gender, place of residence, and history of varicella vaccination. Varicella vaccination history was collected at enrollment by patient (or family) self-report and was not routinely verified against immunization records. Given potential misclassification in this adult cohort, vaccination history was used descriptively. Subsequently, at two critical time points in the disease progression—on the day of initial diagnosis (Day 0) and on the 30th day of follow-up—clinical phenotype indicators are recorded. These include the duration of illness (in days), lesion area (assessed as a percentage of body surface area using the “rule of nines”), pain region (evaluated for distribution using the “rule of nines”), pain intensity (assessed by the numerical rating scale [NRS] ranging from 0 to 10, reflecting the average resting pain over the past 24 hours), and the usage of VZV-related medications (including antiviral drugs, pregabalin, and analgesics).

### 2.3 Detection methods

#### 2.3.1 Virus sample collection

Vesicle fluid samples were collected using two methods, depending on the characteristics of the lesions. For patients with large or multiple vesicles (extending more than 3 cm beyond the lesion margin), the skin was first disinfected with povidone-iodine. A sterile 1-mL syringe was then used to inject a small amount of saline, after which the vesicle fluid was aspirated and transferred into a 1.5-mL Eppendorf tube, followed by storage at -80 °C. For patients with small or few vesicles, a sterile syringe was used to puncture the vesicle, and a sterile swab was gently pressed from one side to the other to absorb the fluid. The swab was then broken off into a 1.5-mL Eppendorf tube and stored at -80 °C. All specimens were labeled with patient ID, name, and date to ensure proper traceability and management.

#### 2.3.2 DNA extraction, identification, and single nucleotide polymorphism analysis

Add 0.5 mL of sterile saline to the collected herpes fluid or swab samples, shake vigorously, and allow to settle before collecting the supernatant. Total DNA is extracted using the TIANamp Virus DNA/RNA Kit (Spin Column, Tiangen Biotech, Cat# DP315-R). After electrophoresis and concentration verification, the DNA is standardized to a concentration of 40–50 ng/μL and stored at -20 °C for future use.

SNP Profiling: Polymerase chain reaction (PCR) amplification and sequencing are performed on the ORF22 region and gE (encoded by ORF68) of VZV. Specific primers are designed for the ORF22 region to amplify a 447 bp fragment that includes five classical SNP sites (37 902, 38 019, 38 055, 38 081, and 38 177), which are utilized for VZV genotype determination. Additionally, specific primers are designed for full-length PCR amplification of the VZV gE gene. The PCR reaction uses a 2× Taq Master Mix (Vazyme, Cat# P112-01) with the following cycling conditions: initial denaturation at 95 °C for 2 min; followed by 30 cycles of denaturation at 95 °C for 1 min, annealing at 50 °C for 1 min, and extension at 72 °C for 45 s; with a final extension at 72 °C for 10 min. The reactions are conducted on a Bio-Rad T100 Thermal Cycler (Bio-Rad T100™ Thermal Cycler, Cat# 1861096), with primer sequences provided in **Supplementary Table 1**. Post-PCR, products are analyzed via electrophoresis on a 1.5% agarose gel. Positive products undergo bidirectional Sanger sequencing by Sangon Biotech (Shanghai, China). Based on sequencing results and nucleotide site genotyping rules in **Supplementary Table 2**, VZV genotyping is performed.

#### 2.3.3 Methods for immunological indicator detection

The levels of VZV gE antigen and its antibody (gEAb), complement components C3 and C4, as well as immunoglobulins IgG and IgM in peripheral blood were quantitatively measured using the enzyme-linked immunosorbent assay (ELISA) method. Peripheral venous blood samples of 4–6 mL were collected from subjects in a fasting state. After allowing the blood to coagulate naturally at room temperature for 20 minutes, it was centrifuged at 2500 rpm for 20 minutes, and the supernatant was extracted for analysis. The ELISA kits used included: VZV gE antigen kit (MyBioSource, Cat# MBS2024185), anti-VZV gE IgG antibody kit (Abcam, Cat# ab108736), complement C3 and C4 kits (Wuhan Fine Biotech, Cat# EH0031 and EH0032), and IgG and IgM kits (Elabscience, Cat# E-EL-H6001 and E-EL-H6003). For all 46 samples, optical density (OD) values were obtained and converted into specific concentrations or quantitative index values based on standard curves provided by the manufacturers. All ELISA experiments were conducted strictly according to the protocols provided in each kit’s manual to ensure accuracy and reproducibility of results.

The expression levels of CD4^+^ T lymphocytes in peripheral blood were assessed via flow cytometry. A 2 mL sample of venous blood was collected and treated with EDTA-K2 anticoagulant (20 IU/mL, Greiner Bio-One, Cat# 454261). Subsequently, 0.1 mL of anticoagulated blood was mixed thoroughly with 20 μL of FITC-labeled CD4 monoclonal antibody (BioLegend, Cat# 317408) to prepare a single-cell suspension. Following incubation, data acquisition and analysis were performed using a BD FACSCanto II flow cytometer (BD Biosciences, USA).

### 2.4 Development of a pain prediction model based on the gE indicator (R version)

#### 2.4.1 Study subjects and variable definitions

This study was based on data collected from patients with HZ, incorporating a total of 46 samples. All participants underwent clinical evaluations and serological tests on Day 0 and Day 30. The primary independent variables included: baseline (Day 0) serum gE antigen level (gE, ng/mL), gEAb assignment, serum immune indicators (IgM, IgG, complement C3/C4, CD4^+^ T lymphocyte percentage), clinical assessment variables (pain score NRS, lesion area, pain distribution range), demographic characteristics (gender, age), and duration of illness (days). The primary independent variables included: baseline (Day 0) serum gE antigen level (gE, ng/mL), gE antibody level (gEAb, expressed as a quantitative index value), serum immune indicators. The outcome variables included: the continuous variable ΔNRS (the change in numerical rating scale score from Day 0 to Day 30) and the binary variable “failure of pain relief,” defined as NRS ≥ 3 or a decrease of < 2 points at Day 30.

#### 2.4.2 Data preprocessing and variable standardization

All analyses were conducted in R (v4.2.1). Data import and cleaning were performed using readxl, janitor, dplyr, tidyr and stringr. Samples missing the core outcome (ΔNRS) were excluded prior to modeling. Missingness in the final modeling dataset (n = 46) was low: 9 of 506 predictor entries were missing (1.78%; 46 participants × 11 predictors). Missing values occurred in two immunological variables, CD4+ T-cell count (n = 5, 10.9%) and complement C4 (n = 4, 8.7%), whereas no missing values were present in primary predictors (gE antigen, gEAb) or key clinical variables (baseline NRS, lesion area). Missing values were imputed using the median for continuous variables and the mode for categorical variables (sex). All continuous predictors were standardized using Z-score transformation (mean = 0, s.d. = 1). All continuous predictors (including gE antigen, gEAb, and immune markers) were standardized using Z-score transformation (mean = 0, s.d. = 1). Categorical variables (e.g., gender) were encoded numerically.

#### 2.4.3 Model development and cross-validation

For the continuous outcome ΔNRS, least absolute shrinkage and selection operator (LASSO) regression with L1 regularization was applied using the R package glmnet (cv.glmnet(…, family = “gaussian”, alpha = 1)), with 5-fold cross-validation to automatically select the regularization parameter λ based on minimization of cross-validation error. For the binary outcome “failure of pain relief,” logistic regression with L1 penalty was employed (cv.glmnet(…, family = “binomial”, alpha = 1)), likewise using 5-fold cross-validation to determine the optimal regularization strength. The classification model was evaluated using the area under the receiver operating characteristic curve (ROC AUC) as the primary performance metric, and fitting as well as prediction were carried out on the training dataset.

#### 2.4.4 Model evaluation and visualization

For the continuous model, goodness of fit and prediction error were assessed using coefficient of determination (R^2^), mean absolute error (MAE), and root mean square error (RMSE). For the classification model, evaluation metrics included the ROC AUC using the pROC package, the precision–recall (PR) curve and PR-AUC using precrec, the Brier score (calculated as mean[(p − y)^2^]), as well as accuracy, precision, recall, and F1 score as needed. To assess probability calibration, a logistic calibration model was built with rms::lrm, and bootstrap calibration curves were generated using rms::calibrate, displaying both the ideal line and the bias-corrected curve. Clinical utility was evaluated by Decision Curve Analysis (DCA) with the rmda package (decision_curve and plot_decision_curve), comparing model strategies with “treat-all” and “treat-none” approaches across different probability thresholds to estimate net benefit. Visualization was performed using ggplot2, pROC, precrec, rms, and rmda.

#### 2.4.5 Model interpretation and scoring system construction

In the LASSO and logistic regression models, the standardized coefficients of the final variables included were extracted to construct a linear scoring card. Taking the variable with the maximum absolute coefficient as the baseline of 10 points, the remaining variables were linearly mapped to a range of 0–10 points. This approach quantifies each variable’s relative contribution to outcome prediction and enables clinicians to perform rapid risk assessment even when computational modeling is not available.

#### 2.4.6 Construction of the ΔNRS predictive model

Using the 30-day ΔNRS as a continuous dependent variable, a LASSO regression model was developed. Candidate independent variables included 11 baseline features: baseline gE level, gEAb assignment, immunological markers (IgM, IgG, C3, C4, CD4^+^ percentage), baseline NRS, pain area, age, and duration of illness (days). All variables were standardized with Z-score normalization, and missing values were imputed using the median. A five-fold cross-validation procedure was employed to select the regularization parameter and identify key predictors. Results were presented as a standardized coefficient bar chart, illustrating each variable’s relative contribution to ΔNRS.

#### 2.4.7 Nested model comparison: clinical-only versus clinical+gE

To assess the incremental predictive value of gE-related parameters beyond routinely available clinical variables, we constructed and compared two nested models. The clinical baseline model (clinical-only) included age, disease duration at presentation (days), baseline NRS score, and baseline lesion area; the clinical+gE model additionally incorporated gE antigen level (ng/mL) and a prespecified coded gE antibody category. The primary outcome was inadequate pain relief at day 30, defined as NRS ≥ 3 at day 30 or a decrease in NRS of < 2 points from baseline (coded as 1 for failure and 0 for success). Both models were fitted using L1-penalized (LASSO) logistic regression; continuous predictors were mean-centered and standardized, and the gE antibody variable was entered as a categorical factor. The penalty parameter λ was selected via 5-fold cross-validation using AUC as the optimization criterion. Model discrimination was quantified by AUC with 95% confidence intervals, and overall prediction error by the Brier score; differences in AUC between models were compared using the DeLong test (two-sided *p* < 0.05).

#### 2.4.8 Minimalist logistic model and sensitivity analysis

To verify the independent contribution of gE-related indicators to the outcome of “failure of pain relief,” a minimalist logistic regression model was constructed, including only gE, gEAb assignment, and two basic clinical variables (age and baseline NRS). The model was fitted using L1 regularization with five-fold cross-validation. Evaluation metrics comprised ROC AUC, PR-AUC, Brier score, calibration curve, and DCA. Based on the standardized coefficients, a 0–10 point scoring chart was created for the minimalist model, serving both as a sensitivity analysis of the main model and as a simplified clinical tool for rapid risk assessment.

### 2.5 Statistical analysis

All analyses were performed in GraphPad Prism 10 and R (v4.2.1). Group comparisons were conducted using paired or unpaired t tests, Mann–Whitney U tests, χ^2^ tests or Fisher’s exact tests, as appropriate. Continuous variables are reported as mean ± standard deviation ( $\bar{\text{x}}\pm \text{s}$) or median with interquartile range [M(IQR), and categorical variables as n (%). All tests were two-sided with *p* < 0.05 considered significant.

Predictive modeling used L1-regularized regression (LASSO/penalized logistic regression) with five-fold cross-validation to select the penalty parameter. Model discrimination was assessed by ROC AUC and PR-AUC, overall prediction error by the Brier score, and calibration and clinical utility by calibration curves and decision curve analysis. The full R workflow is provided in shingles_pain_modeling.R.

## 3 Results

### 3.1 Case characteristics

A total of 52 clinical specimens of VZV, all obtained from vesicular fluid, were collected from outpatients diagnosed with herpes zoster in Chongqing. Of these, 46 samples were successfully sequenced and included in the final analysis. These 46 patients were from 17 different regions of Chongqing.

Among the 46 analyzed patients, 29 were male (63.0%) and 17 were female (37.0%). The patients’ ages ranged from 22 to 79 years, with a median age of 55 years (IQR, 39–71). Baseline characteristics of the analyzed cohort are presented in **Table 1**.

**Table 1:** Baseline characteristics of the 46 patients included in the final analysis.

Characteristics	Male	Female	*P value*
n	29	17	
Age, median (IQR, Q1–Q3)	55 (IQR 39–71)	49 (IQR 33–66))	0.608
History of varicella, n (%)			0.941
Yes	5 (10.9%)	2 (4.3%)	
No	24 (52.2%)	15 (32.6%)	
History of varicella vaccination, n (%)			0.376
Yes	21 (45.7%)	15 (32.6%)	
No	8 (17.4%)	2 (4.3%)	
Disease duration at first diagnosis (days), median (IQR)	5 (4, 6)	5 (4, 6)	0.508
Skin lesion area (Rule of Nines, %) at day 0, mean ± SD	1.6552 ± 0.82531	1.5412 ± 1.0266	0.682
Pain area (Rule of Nines, %) at day 0, mean ± SD	2.4552 ± 1.1706	2.3412 ± 0.85225	0.728
NRS score at day 0, median (IQR)	4 (3, 6)	3 (3, 5)	0.159
Pregabalin daily dose (mg) at day 0, n (%)			0.674
150	22 (47.8%)	12 (26.1%)	
300	1 (2.2%)	1 (2.2%)	
75	1 (2.2%)	2 (4.3%)	
0	5 (10.9%)	2 (4.3%)	
Tramadol capsules daily dose (mg) at day 0, median (IQR)	100 (100, 200)	100 (50, 100)	0.299

Self-reported vaccination history may reflect recall bias due to the cohort’s age (median 55 years) and does not imply infection with the vaccine-derived Oka strain. ORF22 SNP-based genotyping was used for clade assignment and does not distinguish vaccine-related from wild-type viruses within clade 2.

Regarding medical history, 7 of 46 patients (15.2%) reported a history of chickenpox, including 5 males and 2 females, whereas 36 of 46 patients (78.3%) reported prior varicella vaccination, including 21 males and 15 females. Varicella vaccination history was self-reported by patients or their family members.

All included patients sought medical attention soon after the onset of skin lesions or pain symptoms. The median disease duration at first diagnosis was 5 days (IQR, 4-6), with no significant difference between males and females (*p* = 0.508).

In terms of clinical scoring, the mean skin lesion area score at day 0 was 1.60 ± 0.90, the mean pain area score was 2.42 ± 1.05, and the median NRS pain score was 4 (IQR, 3-6). No significant sex-based differences were observed in these measures (all *p* > 0.05).

At the initial diagnosis, the most commonly prescribed pregabalin dose was 150 mg/day (34/46, 73.9%), while the median tramadol dose was 100 mg/day (IQR, 100–200) in males and 100 mg/day (IQR, 50-100) in females. No significant sex-based differences were observed in medication use (*p* = 0.674 and 0.299, respectively).

The clinical symptoms and signs of the cases met the diagnostic criteria for HZ (**Supplementary Figure 1**).

### 3.2 Significant reduction in lesion scores, pain ratings, and analgesic usage within one month

Among the 52 patients enrolled in the study, all completed the 30-day follow-up. All relevant clinical data were collected by designated personnel to ensure data consistency and comparability. Changes in the clinical condition of the patients are presented in **Supplementary Table 3**. The disease duration was 4.88 ± 1.35 days. The lesion area score significantly decreased from 1.60 ± 0.90 at the initial consultation to 0 at 30 days (*p* < 0.0001). The pain area score showed a significant reduction from 2.42 ± 1.05 to 0.39 ± 0.93 (*p* < 0.0001). The NRS pain score decreased significantly from 4.30 ± 1.99 to 1.33 ± 2.14 (*p* < 0.0001). These results indicate a marked improvement in skin lesions, significant pain relief, and a clear trend of clinical symptom amelioration within one month.

The treatment details of the patients are shown in **Supplementary Table 4**. All patients received antiviral therapy for a duration of 7.89 ± 2.20 days. Regarding medication usage, the initial pregabalin dose was 128.80 ± 66.45 mg/day, which significantly decreased to 55.43 ± 94.12 mg/day by day 30 (*p* < 0.0001). The initial tramadol dose was 119.57 ± 78.51 mg/day and significantly reduced to 45.65 ± 82.21 mg/day by day 30 (*p* < 0.0001). This suggests that pain control was progressively achieved, resulting in a marked reduction in analgesic usage.

### 3.3 Success rate of specimen sequencing

DNA was extracted from all 52 specimens, of which 46 exhibited DNA concentrations exceeding 20 ng/μL. PCR amplification targeted specific fragments within the ORF22 region and the gE gene of the VZV genome. Utilizing SNP analysis, the isolated VZV was genotyped, and sequencing and analysis of the gE gene were performed, achieving successful outcomes in 46 cases. The success rate for DNA extraction and sequencing from the specimens was 46 out of 52, or 88.46%.

### 3.4 Genotypic analysis of VZV specimens

The analysis of SNPs within the ORF22 segment identified five VZV genotypes (**Supplementary Table 5**). The results indicated that Clade 2 (Japanese-type [J-type]) was the predominant genotype, accounting for 31 cases, which constituted 67.4% of the sequenced samples. The distribution of the remaining genotypes is as follows: Clade 3 (E2-type) with 7 cases (15.2%), Clade 4 (M2-type) with 5 cases (10.9%), Clade 1 (E1-type) with 2 cases (4.3%), and Clade 5 (M1-type) with 1 case (2.2%).

The mutation characteristics of each genotype are as follows: Clade 1 (E1) genotypes harbored an antisense mutation (A→C) at nucleotide position 38081. Clade 2 (J-type) strains exhibit multiple site mutations at positions 38055, 38081, and 38177, with a total of 5 antisense mutations (T→C, A→G) and 6 synonymous mutations (A→T, C→G). Clade 3 (E2) strains feature 4 antisense mutations (A→C, T→C, A→G) at positions 38055 and 38081, along with 6 deletion mutations detected at position 38229. No identifiable SNP mutations were detected in Clade 4 (M2) and Clade 5 (M1) strains. Among the 46 successfully sequenced samples, 22 samples contained mutated strains, resulting in a mutation rate of 47.8% (22/46).

The findings suggest that Clade 2 (J-type) was the predominant circulating strain among VZV infections in outpatient HZ patients in Chongqing, exhibiting a relatively high mutation frequency.

### 3.5 Analysis of VZV gE gene mutation characteristics

Using the Dumas reference strain as a control, we performed a comparative analysis of the gE gene sequences from 46 clinical isolates of VZV (**Supplementary Table 6**). The results revealed that within the amino acid coding region spanning positions 250-291, 31 samples (67.4%) exhibited a consistent mutation characterized by a T to I substitution, indicating a missense mutation. This suggests that this region may represent a relatively high-frequency mutation hotspot. In addition to these common mutations, several other amino acid coding mutations and missense mutations were observed, including at position 346 (S to A), position 447 (L to V), position 1264 (A to T), position 426 (L to V), position 1309 (A to T), and position 452 (L to V). The average number of nucleotide mutations in the gE gene was 13.46 ± 8.07 (n = 46), with an average number of nucleotide deletions of 5.00 ± 3.71 (n = 46). This indicates a certain degree of genetic variation in the gE gene among different VZV strains.

### 3.6 Analysis of serological markers detection

This study assessed the levels of VZV gE and gEAb, complement components C3 and C4, as well as IgG and IgM, in the peripheral blood of early-stage HZ patients (**Supplementary Table 7**). The results indicated that VZV gE antigen and its specific antibody were detectable in patients’ peripheral blood, with gE levels measuring 110.82 ± 89.91 ng/mL and gEAb values at 1.61 ± 0.49. This suggests that the body has mounted a humoral immune response during active VZV infection. Complement measurements showed that C3 levels were 1.25 ± 0.60 g/L and C4 levels were 0.24 ± 0.12 g/L, both within their respective reference ranges (C3: 0.79-1.52 g/L; C4: 0.16-0.38 g/L). Regarding immunoglobulins, the concentration of IgM was 1.19 ± 1.10 mg/mL, within the normal reference range (0.46-3.04 mg/mL), while IgG levels were elevated at 16.35 ± 7.15 mg/mL, exceeding the normal upper limit (15.60 mg/mL). Flow cytometry results indicated that CD4^+^ T cell levels were 0.28 ± 0.07, remaining within the normal range, suggesting that cellular immune function in early-stage HZ patients is generally intact.

In summary, the results demonstrate that VZV gE antigen and gEAb can be detected at the early stages of HZ onset. Although immunoglobulin and complement levels are mostly within reference ranges, some indicators, such as IgG, are significantly elevated, while CD4^+^ T cell counts remain stable. This suggests that the immune system is actively responding to VZV infection. These immunological characteristics align with the typical biological manifestations of early-stage HZ, providing a foundation for further investigation into viral clearance and subsequent immune recovery.

### 3.7 A predictive model for poor pain relief outcomes integrating gE markers and clinical and immunological features

This study developed a multivariable L1-regularized logistic regression model based on complete follow-up data from 46 patients with HZ. Input variables included baseline gE level, gEAb assignment, immunological indicators (IgM, IgG, C3, C4, CD4^+^), and clinical features (age, duration of illness, baseline NRS score, lesion area, and pain distribution range). The binary outcome was the presence of persistent moderate-to-severe pain at 30 days (defined as NRS ≥ 3 or a decrease of < 2 points). Individual prediction probabilities were obtained through five-fold cross-validation.

**Figure 2:**
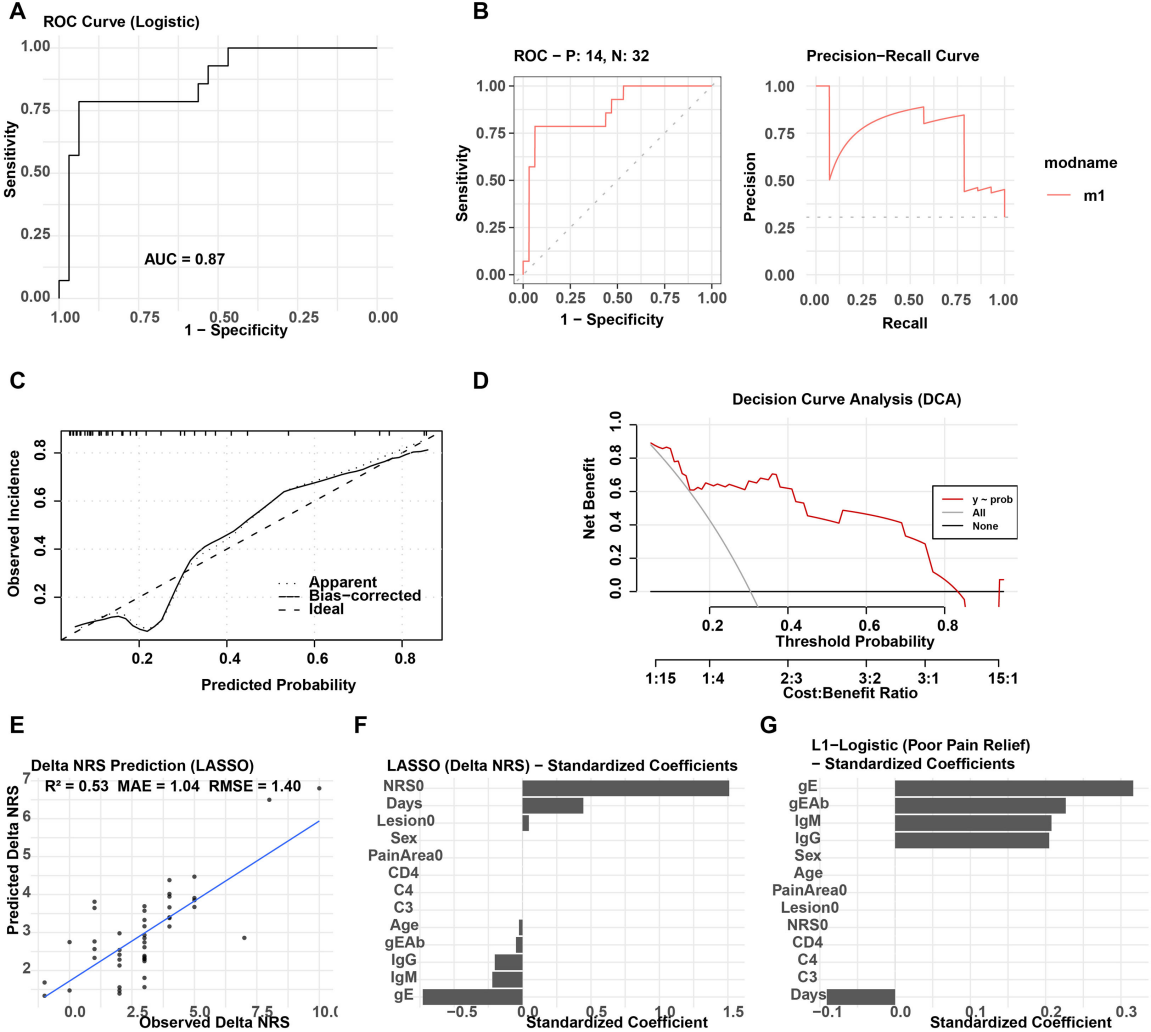
Comprehensive evaluation of pain outcome prediction models (Logistic and LASSO). **(A)** ROC curve of the L1-penalized logistic regression model predicting persistent moderate-to-severe pain at day 30 (binary outcome); **(B)** ROC and PR curves based on cross-validated predicted probabilities; **(C)** Calibration plot showing agreement between predicted and observed probabilities; **(D)** DCA illustrating net benefit across a range of threshold probabilities; **(E)** Scatter plot of observed vs. predicted ΔNRS values using LASSO regression, with performance metrics annotated; **(F)** Standardized coefficients from the LASSO model predicting ΔNRS (continuous outcome); **(G)** Standardized coefficients from the L1-penalized logistic regression model predicting poor pain relief. All models used Z-score standardized predictors and five-fold cross-validation for tuning of the regularization parameter; performance metrics are reported as internal estimates based on the study cohort (n=46).

The model showed good discriminative performance, with an AUC of 0.87 on the ROC curve (**Figure 2A**) and maintained high precision in the high-recall range (**Figure 2B**). Calibration of predicted probabilities was satisfactory (**Figure 2C**), with a Brier score of 0.14. DCA demonstrated that the model provided greater net benefit across most threshold probabilities compared with either a “treat-all” or “treat-none” strategy (**Figure 2D**). In addition, **Figure 2G** displays the standardized coefficients from the L1-logistic model, illustrating the contribution of each variable to outcome prediction.

To further construct a predictive model for a continuous outcome, ΔNRS (from Day 0 to Day 30) was used as the dependent variable and modeled with LASSO regression. Model performance was evaluated with R^2^ = 0.53, MAE = 1.04, and RMSE = 1.40 (**Figure 2E**). **Figure 2F** presents the standardized coefficients of the selected variables, indicating that gE, gEAb, and IgM exert strong explanatory power for changes in NRS scores.

To assess the incremental predictive value of gE-related parameters beyond routinely available clinical variables, we compared two nested models. The clinical-only model included age, disease duration (days), baseline NRS score, and baseline lesion area. Adding gE antigen level and the prespecified coded gE antibody variable yielded improved discrimination (AUC 0.79 vs 0.86) and lower prediction error (Brier score 0.166 vs 0.126). The absolute AUC difference was ΔAUC = 0.07, and the DeLong test did not reach statistical significance (*p* = 0.144). Overall, adding gE-related markers was associated with numerically improved discrimination and lower prediction error beyond clinical predictors alone, although the AUC difference was not statistically significant in this sample (**Supplementary Figure 2**).

### 3.8 Secondary modeling and sensitivity analysis results of gE-related indicators

To further verify the independence and stability of gE in pain prediction, a minimalist logistic regression model was constructed that included only gE, gEAb assignment, and two basic clinical variables (age and baseline NRS score) to predict the presence of persistent moderate-to-severe pain at 30 days. The model was fitted using L1 regularization with five-fold cross-validation, and its predictive performance is shown in **Supplementary Figure 3**. The model achieved an AUC of 0.87 on the ROC curve (**Supplementary Figure 3A**), demonstrating strong discriminative ability. The PR-AUC likewise indicated good precision in the high-recall range (**Supplementary Figure 3B**). Calibration analysis showed close agreement between predicted probabilities and observed outcomes, with a Brier score of 0.12 (**Supplementary Figure 3C**). DCA revealed that across most probability thresholds, the model provided a greater net benefit than either the “treat-all” or “treat-none” strategy (**Supplementary Figure 3D**). In the final model, only gE, gEAb, and age were retained, while the baseline NRS variable was excluded (coefficient = 0). The scoring chart (**Supplementary Figure 3E**) indicates that gE received the highest weight (10 points), gEAb approximately 6.8 points, and age a lower score, underscoring the dominant predictive value of gE-related indicators in this minimalist model. An exploratory baseline serum gE antigen cut-off for classifying poor pain relief at day 30 was derived using ROC analysis (Youden index). The optimal threshold was 132.5 ng/mL (sensitivity 81.8%, specificity 85.7%). This cut-off supports within-cohort risk stratification and requires external validation.

## 4 Discussion

VZV lineage typing in an HZ outpatient cohort from Chongqing showed clade 2 (J-type) predominance (76.1%), consistent with reports from other East Asian settings (17). ORF22 SNP-based typing allows assignment of VZV clades, but it does not distinguish vaccine (vOka)-related strains from wild-type viruses within clade 2. Therefore, the predominance of clade 2 in this cohort should be interpreted as a regional lineage pattern rather than as evidence of vaccine-strain reactivation. In the present study, some participants reported prior varicella vaccination despite no clear recalled history of chickenpox. Because vaccination and prior infection histories were self-reported, recall bias or misclassification cannot be excluded, particularly in this adult cohort. In addition, because breakthrough varicella after vaccination can be mild or unrecognized, a reported history of vaccination without a clear recalled history of chickenpox does not exclude the possibility of subsequent reactivation of a wild-type virus acquired after vaccination. Definitive discrimination between vOka-related and wild-type viruses would require analysis of vaccine-informative loci (e.g., ORF62 SNPs) or broader sequencing (18). Accordingly, vaccination history in this study was interpreted descriptively.

At the gE locus, extensive variation was observed, including a frequent Thr-to-Ile substitution clustered within aa 250–291 and a substantial burden of mutations and deletions, suggesting a characteristic local mutation spectrum. gE is a type I membrane glycoprotein whose ectodomain constitutes a major antigenic surface and mediates key host–virus interactions (19). The aa 250–291 hotspot lies within the N-terminal ectodomain and within an epitope-enriched segment (codons 39–344) previously used for gE epitope mapping across clinical isolates (20). Recent structural work indicates multiple conformational antigenic sites on gE, with vaccine-elicited human antibodies preferentially targeting the gI-interacting face; substitutions clustered immediately upstream of the flexible ~295–305 region may therefore influence local dynamics and epitope accessibility rather than global folding (19).

Immunologically, early serum detection of both gE antigens and antibodies was possible, with significantly elevated IgG levels, while complement (C3, C4) and CD4^+^ T cell levels remained within normal ranges, indicating activation of humoral immunity while maintaining cellular immune function. Clinical follow-up revealed a significant reduction in lesion area, pain extent, and NRS scores within 30 days; however, some patients experienced poor pain relief. A predictive model constructed based on gE and immunological markers demonstrated high discriminative power (AUC = 0.87, PR-AUC = 0.74). These findings not only provide the first systematic molecular epidemiological evidence for the Chongqing region but also offer new support for gE as a clinical biomarker, highlighting the study’s significance and innovation.

Compared with prior literature, our findings are consistent in several respects. Clade 2 (J-type) predominated in Chongqing, in line with reports from Japan, Korea and eastern China, supporting a largely stable East Asian lineage background (21, 22). This observation also fits the broader epidemiological landscape in China, where ORF22-based surveillance and provincial datasets indicate nationwide clade 2 dominance with only sporadic detection of other clades, consistent with the low-frequency non–clade 2 lineages in our cohort (17, 23–25). In contrast, gE polymorphism appears more geographically structured: multiple variable sites have been described across regions, whereas we observed a high-frequency hotspot at aa 250–291, differing from some European patterns and suggesting region-specific evolutionary pressures (26, 27). Immunologically, the acute-phase IgG increase reported previously was reproduced in our cohort (28, 29), while CD4+ T-cell levels remained within reference ranges; differences in age structure, baseline immune status and viral genetic background may contribute to variability in cellular immune findings across studies. This broad geographic concordance suggests limited regional stratification of major VZV lineages within China, and supports the epidemiological representativeness of our Chongqing cohort for southwest China.

Early detectability of circulating gE in acute HZ, together with its association with subsequent pain relief, suggests that viral antigen burden may shape pain trajectories. This extends prior work that has emphasized cellular immunity as the main constraint on VZV reactivation, with less focus on viral glycoproteins as correlates of clinical pain phenotypes (30). A plausible mechanism is that gE variation modulates immunogenicity and the magnitude or quality of the humoral response, thereby influencing inflammatory and neuroinflammatory signalling relevant to pain persistence. In support, L447V maps to the C-terminal ectodomain within an Fc-binding-domain–annotated region (~301–516); although Leu-to-Val is conservative, subtle changes on exposed surfaces may alter conformational epitope geometry and contribute to inter-individual variability in gEAb levels and pain outcomes (19). Collectively, these observations support an integrative framework linking viral genetic variation, gE-driven immune responses and pain phenotype.

Clinically, gE and immune markers may enable early risk stratification in HZ. Models incorporating these variables showed good discrimination for poor pain relief, supporting their potential utility for identifying patients who may benefit from closer follow-up and earlier escalation of antiviral and analgesic management, with consideration of adjunctive immune-modulating strategies where appropriate. Across multiple modelling specifications, gE-related variables remained consistently informative, underscoring the robustness of their association with pain outcomes in this cohort. Beyond prognostication, the immunogenicity of gE also supports its relevance to future vaccine refinement and immune-targeted interventions.

This study has several limitations. First, the sample size was modest (52 cases; 46 successfully sequenced) and the number of binary outcome events was limited, reducing power and generalizability and increasing the risk of over-parameterization with many covariates. L1 regularization, nested model comparisons to quantify the incremental value of gE, and a parsimonious model were used to improve shrinkage and stability, but performance estimates remain internal and require external validation and calibration. Second, the cohort was single-center and outpatient-based, with no hospitalized cases or immunosuppressed populations; exclusion of malignancy, autoimmune disease and current immunosuppressive therapy may introduce selection bias and limits applicability to high-risk groups. Third, varicella vaccination history was self-reported and not routinely verified, and may be misclassified in adults, potentially confounding immune-background analyses. Fourth, the genotyping loci were optimized for clade assignment rather than vaccine-strain identification, preventing direct assessment of vaccine-strain–associated reactivation. Fifth, common comorbidities and immunocompromised status were not collected or quantified in a standardized manner, limiting adjustment for residual confounding. Sixth, immunological measurements were largely single time-point peripheral blood assays with short follow-up, without ganglionic microenvironment data or long-term longitudinal monitoring, limiting inference on immune–viral dynamics over disease evolution.

Future work could proceed in several directions. First, larger multi-centre and multi-regional cohorts, including hospitalized patients and immunosuppressed/multimorbid populations, are needed to confirm lineage distributions, gE variation patterns and the reproducibility of the prediction models, with external validation and calibration. Second, structural biology, epitope mapping and functional assays should be combined to define how key gE mutations affect conformation, immunogenicity, antibody binding and inflammatory/neuroinflammatory pathways. Third, longitudinal cohorts with repeated sampling are required to delineate the temporal relationships among circulating gE antigen/antibody levels, immune markers and pain outcomes, and to define predictive windows relevant to PHN. Fourth, higher-resolution molecular approaches incorporating vaccine-informative loci or broader sequencing would improve discrimination of vOka versus wild-type strains and enable more rigorous phenotype–genotype analyses; in parallel, gE-based vaccine refinement and immune-targeted interventions warrant exploration to support personalized HZ prevention and management.

## 5 Conclusion

In summary, we can draw the following preliminary conclusions: This study systematically reveals the distribution characteristics of VZV gene types and the high-frequency mutation patterns of the gE gene in outpatient HZ patients. Notably, a concentrated distribution of T→I missense mutations was observed in the amino acid region 250-291, suggesting this region may be a functional hotspot. The gE protein and its antibodies can be detected early in the disease course, and gE levels are significantly associated with subsequent pain relief outcomes. A logistic prediction model constructed based on gE indicators, immune parameters, and clinical characteristics demonstrates good discriminative ability (AUC = 0.87), effectively identifying high-risk individuals for poor pain relief. The findings indicate that gE is not only a key molecular site for VZV variation but also an important potential biomarker for predicting HZ pain outcomes.

## Data Availability

The original contributions presented in the study are included in the article/**Supplementary Material**. Further inquiries can be directed to the corresponding author.

## Supplementary material

Supplementary Figure 1 Representative images of skin lesions and vesicle fluid collection from patients with HZ.

Supplementary Figure 2 Comparison of ROC curves for predicting poor pain relief with clinical variables alone versus clinical plus gE markers.

Supplementary Figure 3 Predictive performance and variable weighting of the simplified logistic regression model. (A) ROC curve (AUC = 0.87) evaluating the model’s ability to discriminate poor pain-relief outcomes; (B) PR curve assessing prediction accuracy in the high-recall range; (C) Calibration curve showing agreement between predicted probabilities and observed event rates, with a Brier score of 0.12; (D) DCA demonstrating greater net benefit of the model compared with “treat-all” or “treat-none” strategies across a range of probability thresholds; (E) Variable scoring chart mapping standardized coefficients to a 0–10 scale: gE received the highest score (10), gEAb scored 6.85, Age scored 0.44, and NRS0 was not included in the final model (score 0).
