# Supplementary figures and images for: gE mutations and VZV genotypes jointly predict pain relief outcomes in herpes zoster: an integrative immunologic and modeling study
Source: Front Immunol. 2026 Apr 29;17:1715267. doi: 10.3389/fimmu.2026.1715267 (PMC13168172; doi:10.3389/fimmu.2026.1715267)

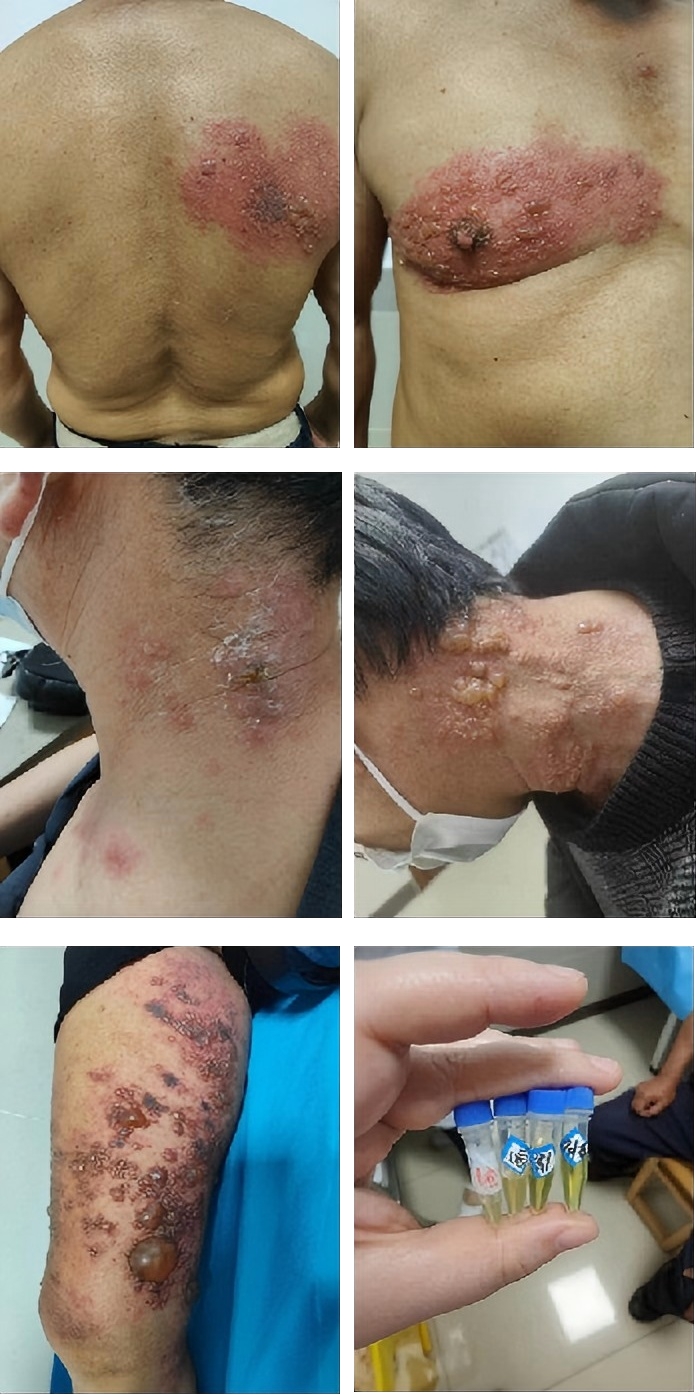

Supplement: Supplementary Figure 1 — Representative images of skin lesions and vesicle fluid collection from patients with HZ. [file Image1.jpeg]

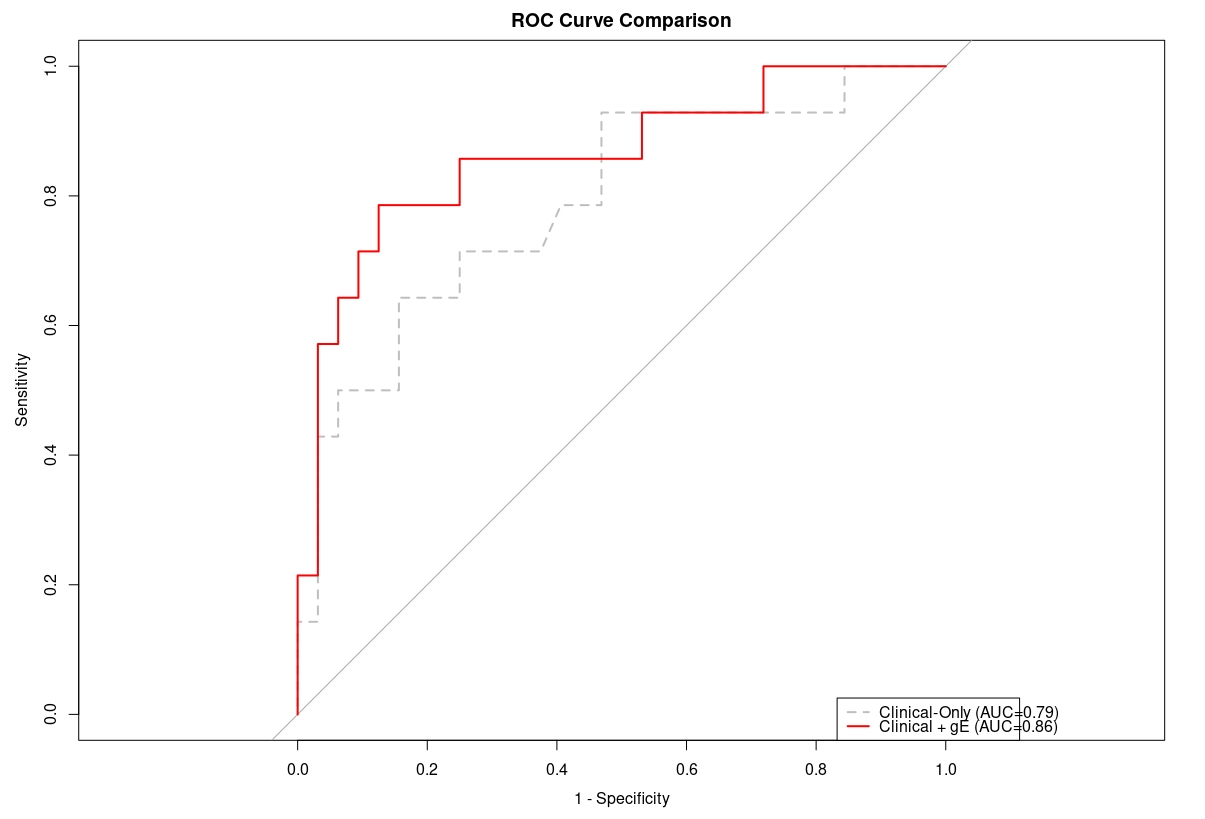

Supplement: Supplementary Figure 2 — Comparison of ROC curves for predicting poor pain relief with clinical variables alone versus clinical plus gE markers. [file Image2.jpeg]

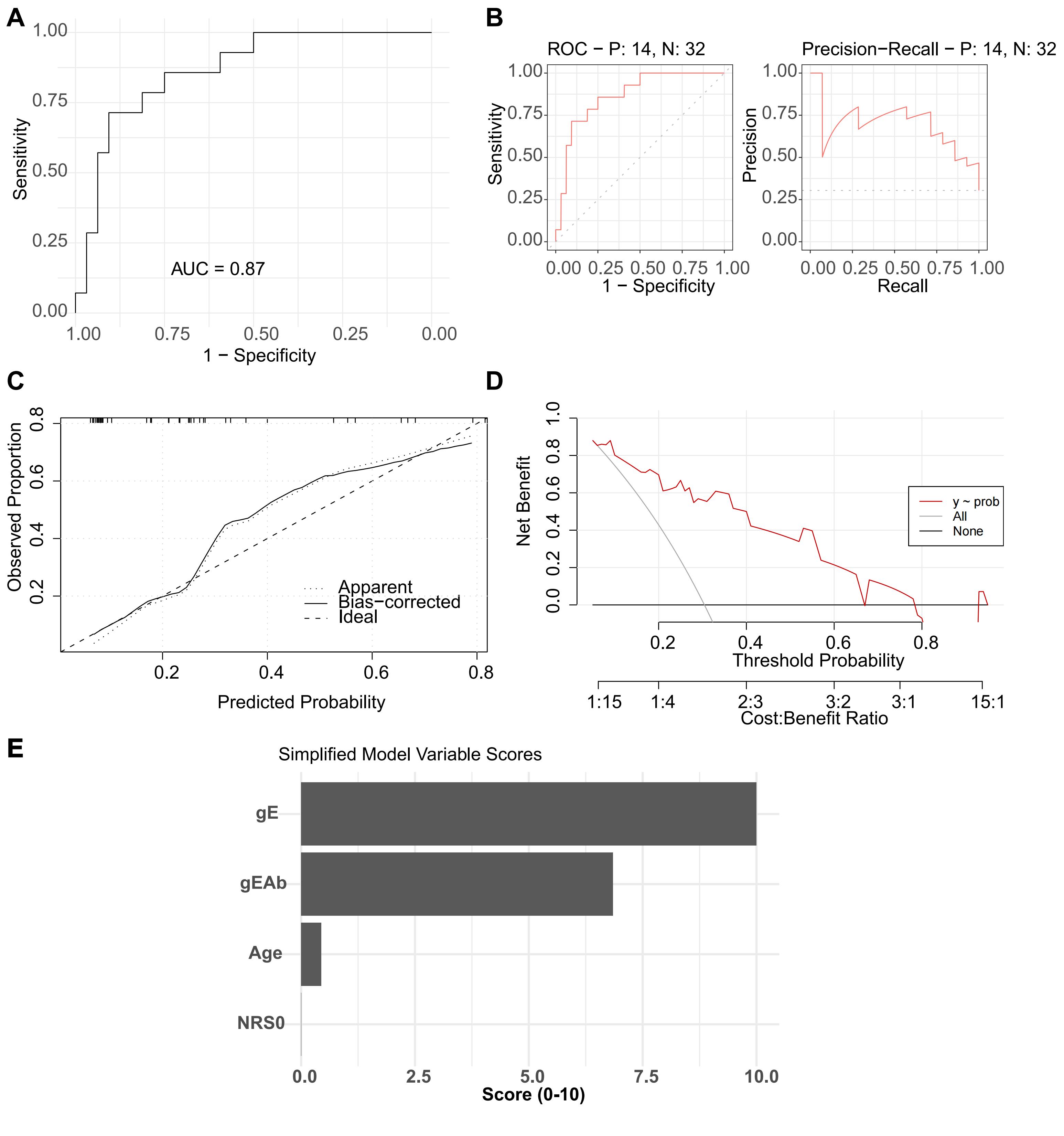

Supplement: Supplementary Figure 3 — Predictive performance and variable weighting of the simplified logistic regression model. (A) ROC curve (AUC = 0.87) evaluating the model’s ability to discriminate poor pain-relief outcomes; (B) PR curve assessing prediction accuracy in the high-recall range; (C) Calibration curve showing agreement between predicted probabilities and observed event rates, with a Brier score of 0.12; (D) DCA demonstrating greater net benefit of the model compared with “treat-all” or “treat-none” strategies across a range of probability thresholds; (E) Variable scoring chart mapping standardized coefficients to a 0–10 scale: gE received the highest score (10), gEAb scored 6.85, Age scored 0.44, and NRS0 was not included in the final model (score 0). [file Image3.jpeg]
