# Supplementary material for: gE mutations and VZV genotypes jointly predict pain relief outcomes in herpes zoster: an integrative immunologic and modeling study
Source: Front Immunol. 2026 Apr 29;17:1715267. doi: 10.3389/fimmu.2026.1715267 (PMC13168172; doi:10.3389/fimmu.2026.1715267)
Supplement: Supplementary file 4 [file Table1.docx]

**Table S1. PCR Primer sequences for VZV gE gene.**

| **Primer** | **Sequence (5′-3′)** | **Position (Dumas)** |
| --- | --- | --- |
| VgE-F1 | GGCGTTTTATTTAGCGTTTG | 115599-115618 |
| VgE-R1 | ACATCCACCACCACGTCTTG | 115620-115601 |
| VgE-F2 | GCATTGAGGTGTCAGTGGAAG | 116320-116339 |
| VgE-R2 | CCAACAGTGTATGCTAGGGCT | 117287-117268 |
| VgE-F3 | CTTTACCTGCGCCATATTAGC | 117050-117069 |
| VgE-R3 | CACTTCCGACTATTGGATTGT | 100734-117925 |
