# Supplementary material for: gE mutations and VZV genotypes jointly predict pain relief outcomes in herpes zoster: an integrative immunologic and modeling study
Source: Front Immunol. 2026 Apr 29;17:1715267. doi: 10.3389/fimmu.2026.1715267 (PMC13168172; doi:10.3389/fimmu.2026.1715267)
Supplement: Supplementary file 5 [file Table2.docx]

**Table S2. VZV genotyping rules.**

| **VZV strain** | **Genotype** | **37902** | **38019** | **38055** | **38081** | **38177** | **38229** |
| --- | --- | --- | --- | --- | --- | --- | --- |
| Dumas | E1 (Clade 1) | A | G | T | A | G | A |
| Oka parental | J (Clade 2) | G | G | C | C | A | A |
| California 123J | M1 (Clade 5) | A | G | T | C | G | G |
| Morocco 1 | M2 (mor) (Clade 4) | A | G | C | C | A | G |
| Mexico 68 | M2 (mex) (Clade 4) | A | A | C | C | A | A |
| Iceland 006 | M2 (ice) (Clade 4) | A | G | C | A | G |  |
|  | E2 (Clade 3) | A | G | T | A | G |  |
