# Supplementary material for: gE mutations and VZV genotypes jointly predict pain relief outcomes in herpes zoster: an integrative immunologic and modeling study
Source: Front Immunol. 2026 Apr 29;17:1715267. doi: 10.3389/fimmu.2026.1715267 (PMC13168172; doi:10.3389/fimmu.2026.1715267)
Supplement: Supplementary file 6 [file Table3.docx]

**Table S3. Patient condition related indicators.**

| **Observation indicators** | **Disease duration (d)** | **Skin lesion area (nine point scale, %)** | | **Pain area (nine point scale, %)** | | **NRSscore** | |
| --- | --- | --- | --- | --- | --- | --- | --- |
| Time |  | 0d | 30d | 0d | 30d | 0d | 30d |
| Values | 4.88 ± 1.35 | 1.6 ± 0.90 | 0 ± 0 | 2.42 ± 1.05 | 0.39 ± 0.93 | 4.30 ± 1.99 | 1.33 ± 2.14 |
| *P-value* |  | <0.0001 | | <0.0001 | | <0.0001 | |
