# Supplementary material for: gE mutations and VZV genotypes jointly predict pain relief outcomes in herpes zoster: an integrative immunologic and modeling study
Source: Front Immunol. 2026 Apr 29;17:1715267. doi: 10.3389/fimmu.2026.1715267 (PMC13168172; doi:10.3389/fimmu.2026.1715267)
Supplement: Supplementary file 7 [file Table4.docx]

**Table S4. Patient medication treatment data.**

| **Observation indicators** | **Antiviral treatment duration (d)** | **Pregabalin (mg/d)** | | **Tramadol Capsules (mg/d)** | |
| --- | --- | --- | --- | --- | --- |
| Time |  | 0d | 30d | 0d | 30d |
| Values | 7.89 ± 2.20 | 128.80 ± 66.45 | 55.43 ± 94.12 | 119.57 ± 78.51 | 45.65 ± 82.21 |
| *P-value* |  | <0.0001 |  | <0.0001 |  |
