# Supplementary material for: gE mutations and VZV genotypes jointly predict pain relief outcomes in herpes zoster: an integrative immunologic and modeling study
Source: Front Immunol. 2026 Apr 29;17:1715267. doi: 10.3389/fimmu.2026.1715267 (PMC13168172; doi:10.3389/fimmu.2026.1715267)
Supplement: Supplementary file 8 [file Table5.docx]

**Table S5.** **VZV sample collection information and genotype.**

| **Sample number** | **VZV genotype** | **History of chickenpox (yes: 1, no: 2)** | **History of chickenpox vaccination (yes: 1, no: 2)** | **Place of long-term residence** |
| --- | --- | --- | --- | --- |
| 1 | Clade 2 | 2 | 1 | Chongqing |
| 2 | Clade 2 | 2 | 1 | Chongqing |
| 3 | Clade 3 | 1 | 1 | Chongqing |
| 4 | Clade 2 | 2 | 1 | Chongqing |
| 5 | Clade 1 | 2 | 1 | Chongqing |
| 6 | Clade 4 | 2 | 2 | Chongqing |
| 7 | Clade 4 | 2 | 2 | Chongqing |
| 8 | Clade 2 | 2 | 1 | Chongqing |
| 9 | Clade 2 | 1 | 1 | Chongqing |
| 10 | Clade 2 | 2 | 2 | Chongqing |
| 11 | Clade 2 | 2 | 1 | Chongqing |
| 12 | Clade 2 | 2 | 1 | Chongqing |
| 13 | Clade 1 | 2 | 1 | Chongqing |
| 14 | Clade 4 | 2 | 1 | Chongqing |
| 15 | Clade 2 | 2 | 1 | Chongqing |
| 16 | Clade 2 | 1 | 1 | Chongqing |
| 17 | Clade 3 | 2 | 1 | Chongqing |
| 18 | Clade 2 | 2 | 2 | Chongqing |
| 19 | Clade 3 | 1 | 1 | Chongqing |
| 20 | Clade 2 | 2 | 1 | Chongqing |
| 21 | Clade 2 | 2 | 2 | Chongqing |
| 22 | Clade 2 | 2 | 2 | Chongqing |
| 23 | Clade 2 | 2 | 1 | Chongqing |
| 24 | Clade 2 | 2 | 1 | Chongqing |
| 25 | Clade 3 | 2 | 2 | Chongqing |
| 26 | Clade 3 | 2 | 1 | Chongqing |
| 27 | Clade 2 | 2 | 1 | Chongqing |
| 28 | Clade 2 | 2 | 1 | Chongqing |
| 29 | Clade 4 | 2 | 1 | Chongqing |
| 30 | Clade 2 | 1 | 2 | Chongqing |
| 31 | Clade 2 | 2 | 1 | Chongqing |
| 32 | Clade 2 | 2 | 1 | Chongqing |
| 33 | Clade 2 | 1 | 1 | Chongqing |
| 34 | Clade 2 | 2 | 1 | Chongqing |
| 35 | Clade 3 | 2 | 1 | Chongqing |
| 36 | Clade 2 | 2 | 1 | Chongqing |
| 37 | Clade 2 | 2 | 1 | Chongqing |
| 38 | Clade 2 | 2 | 2 | Chongqing |
| 39 | Clade 5 | 2 | 1 | Chongqing |
| 40 | Clade 4 | 2 | 2 | Chongqing |
| 41 | Clade 2 | 2 | 1 | Chongqing |
| 42 | Clade 2 | 2 | 1 | Chongqing |
| 43 | Clade 2 | 2 | 1 | Chongqing |
| 44 | Clade 3 | 2 | 1 | Chongqing |
| 45 | Clade 2 | 2 | 1 | Chongqing |
| 46 | Clade 2 | 2 | 1 | Chongqing |

Note: Vaccination history was obtained by patient (or family) self-report and was not routinely verified against immunization records. Given potential misclassification in this adult cohort (for example, recall error or conflation with childhood varicella infection), vaccination history was used descriptively
