# Supplementary material for: gE mutations and VZV genotypes jointly predict pain relief outcomes in herpes zoster: an integrative immunologic and modeling study
Source: Front Immunol. 2026 Apr 29;17:1715267. doi: 10.3389/fimmu.2026.1715267 (PMC13168172; doi:10.3389/fimmu.2026.1715267)
Supplement: Supplementary file 9 [file Table6.docx]

**Table S6. The gE gene mutation status.**

| **Number** | **Detection element** | **Specific information** | **Specific information** | **Number of base mutations** | **Base deletions** |
| --- | --- | --- | --- | --- | --- |
| 1 | Genetic loci | 284 | 346 | 17 | 12 |
|  | Amino acid mutation | T→I | S→A |  |  |
| 2 | Genetic loci | 291 | 447 | 10 | 7 |
|  | Amino acid mutation | T→I | L→V |  |  |
| 3 | Genetic loci |  |  | 9 | 4 |
|  | Amino acid mutation |  |  |  |  |
| 4 | Genetic loci |  |  | 25 | 18 |
|  | Amino acid mutation |  |  |  |  |
| 5 | Genetic loci | 266 |  | 20 | 6 |
|  | Amino acid mutation | T→I |  |  |  |
| 6 | Genetic loci |  |  | 11 | 5 |
|  | Amino acid mutation |  |  |  |  |
| 7 | Genetic loci |  |  | 9 | 5 |
|  | Amino acid mutation |  |  |  |  |
| 8 | Genetic loci |  |  | 12 | 7 |
|  | Amino acid mutation |  |  |  |  |
| 9 | Genetic loci |  |  | 10 | 6 |
|  | Amino acid mutation |  |  |  |  |
| 10 | Genetic loci |  | 1264 | 15 | 8 |
|  | Amino acid mutation |  | A→T |  |  |
| 11 | Genetic loci | 260 |  | 10 | 7 |
|  | Amino acid mutation | T→I |  |  |  |
| 12 | Genetic loci | 272 |  | 18 | 9 |
|  | Amino acid mutation | T→I |  |  |  |
| 13 | Genetic loci | 272 |  | 22 | 18 |
|  | Amino acid mutation | T→I |  |  |  |
| 14 | Genetic loci |  |  | 7 | 4 |
|  | Amino acid mutation |  |  |  |  |
| 15 | Genetic loci |  |  | 6 | 3 |
|  | Amino acid mutation |  |  |  |  |
| 16 | Genetic loci |  |  | 11 | 6 |
|  | Amino acid mutation |  |  |  |  |
| 17 | Genetic loci | 268 |  | 12 | 8 |
|  | Amino acid mutation | T→I |  |  |  |
| 18 | Genetic loci | 250 |  | 17 | 11 |
|  | Amino acid mutation | T→I |  |  |  |
| 19 | Genetic loci | 260 |  | 15 | 8 |
|  | Amino acid mutation | T→I |  |  |  |
| 20 | Genetic loci |  |  | 10 | 7 |
|  | Amino acid mutation |  |  |  |  |
| 21 | Genetic loci | 286 | 426 | 18 | 9 |
|  | Amino acid mutation | T→I | L→V |  |  |
| 22 | Genetic loci | 275 |  | 23 | 15 |
|  | Amino acid mutation | T→I |  |  |  |
| 23 | Genetic loci |  |  | 7 | 4 |
|  | Amino acid mutation |  |  |  |  |
| 24 | Genetic loci |  |  | 6 | 3 |
|  | Amino acid mutation |  |  |  |  |
| 25 | Genetic loci |  |  | 11 | 6 |
|  | Amino acid mutation |  |  |  |  |
| 26 | Genetic loci | 271 | 1309 | 12 | 8 |
|  | Amino acid mutation | T→I | A→T |  |  |
| 27 | Genetic loci | 239 |  | 17 | 11 |
|  | Amino acid mutation | T→I |  |  |  |
| 28 | Genetic loci |  |  | 15 | 8 |
|  | Amino acid mutation |  |  |  |  |
| 29 | Genetic loci | 273 |  | 10 | 7 |
|  | Amino acid mutation | T→I |  |  |  |
| 30 | Genetic loci | 277 |  | 18 | 9 |
|  | Amino acid mutation | T→I |  |  |  |
| 31 | Genetic loci | 256 |  | 20 | 13 |
|  | Amino acid mutation | T→I |  |  |  |
| 32 | Genetic loci | 264 |  | 7 | 4 |
|  | Amino acid mutation | T→I |  |  |  |
| 33 | Genetic loci |  |  | 6 | 3 |
|  | Amino acid mutation |  |  |  |  |
| 34 | Genetic loci |  |  | 11 | 6 |
|  | Amino acid mutation |  |  |  |  |
| 35 | Genetic loci | 256 |  | 13 | 6 |
|  | Amino acid mutation | T→I |  |  |  |
| 36 | Genetic loci | 290 |  | 19 | 12 |
|  | Amino acid mutation | T→I |  |  |  |
| 37 | Genetic loci | 272 |  | 14 | 8 |
|  | Amino acid mutation | T→I |  |  |  |
| 38 | Genetic loci |  |  | 12 | 9 |
|  | Amino acid mutation |  |  |  |  |
| 39 | Genetic loci | 238 |  | 16 | 10 |
|  | Amino acid mutation | T→I |  |  |  |
| 40 | Genetic loci |  |  | 17 | 12 |
|  | Amino acid mutation |  |  |  |  |
| 41 | Genetic loci |  |  | 9 | 8 |
|  | Amino acid mutation |  |  |  |  |
| 42 | Genetic loci | 283 |  | 5 | 2 |
|  | Amino acid mutation | T→I |  |  |  |
| 43 | Genetic loci | 279 |  | 15 | 9 |
|  | Amino acid mutation | T→I |  |  |  |
| 44 | Genetic loci | 250 | 452 | 15 | 7 |
|  | Amino acid mutation | T→I | L→V |  |  |
| 45 | Genetic loci | 266 |  | 16 | 8 |
|  | Amino acid mutation | T→I |  |  |  |
| 46 | Genetic loci | 261 |  | 21 | 15 |
|  | Amino acid mutation | T→I |  |  |  |
