# Supplementary material for: gE mutations and VZV genotypes jointly predict pain relief outcomes in herpes zoster: an integrative immunologic and modeling study
Source: Front Immunol. 2026 Apr 29;17:1715267. doi: 10.3389/fimmu.2026.1715267 (PMC13168172; doi:10.3389/fimmu.2026.1715267)
Supplement: Supplementary file 10 [file Table7.docx]

**Table S7. Serological indicators of HZ patients.**

| **Detection indicators** | **gE protein level (ng/ml)** | **gEAb (assigned value)** | **IgM (mg/ml)** | **IgG (mg/ml)** | **Complement C3 (g/l)** | **Complement C4 (g/l)** | **CD4 count (expressed in decimal form)** |
| --- | --- | --- | --- | --- | --- | --- | --- |
| Mean | 110.82 | 1.61 | 1.19 | 16.35 | 1.25 | 0.24 | 0.28 |
| SD | 89.91 | 0.49 | 1.10 | 7.15 | 0.60 | 0.12 | 0.07 |

Note: Reference range: IgM (0.46-3.04) g/L; IgG (7.51-15.60) g/L; Complement C3 (0.79-1.52) g/L; Complement C4(0.16-0.38) g/L.
